# A generic part of specific combined responses to biotic and abiotic stresses in crops: Overcoming multifaceted challenges towards new opportunities

**DOI:** 10.3389/fpls.2023.1140808

**Published:** 2023-02-24

**Authors:** Larissa Adamik, Thierry Langin, Ludovic Bonhomme

**Affiliations:** Université Clermont Auvergne, Institut national de recherche pour l'agriculture, l'alimentation et l'environnement (INRAE), Génétique, diversité et ecophysiologie des céréales (GDEC), Clermont-Ferrand, France

**Keywords:** multistress, combined stress, abiotic and biotic challenges, generic responses, crops

## Introduction

Due to climate change, crops are subjected to complex biotic and abiotic environments during their life cycle, with increasingly fluctuating conditions and a recurring set of simultaneous challenges (Eastburn et al., 2011; Thornton et al., 2014; Ray et al., 2019). Compared to isolated stress, the combination of detrimental conditions can result in prominent changes in plant physiology that are difficult to anticipate from current knowledge. Plant responses to co-occurring environmental stresses can be radically different from the known adjustments to an individual stress and even far from the expected additive response to the same stresses occurring separately (Atkinson and Urwin, 2012). This emphasizes that the plant’s response to one constraint depends on its basal physiological status primarily shaped by a multitude of other environmental factors. Considered in breeding programs since decades (Johnson, 1984), this rationale is now clearly recognized and further became even more meaningful with the current challenges of climate change that comes along with increasing constraint combinations. So far, the available knowledge about CS responses was generated with several species including *Arabidopsis thaliana*, and a range of economically important crops. However, the knowledge generated for each genotype is still limited and available data may hardly be transferred within or between species, emphasizing the need of an integrative understanding of the interactions between stresses, especially in crops to secure the needs of a growing global population (e.g. Xu et al., 2008; Campo et al., 2012; Rejeb et al., 2014; Tani et al., 2018). A thorough understanding specifically in crops is a prerequisite to the identification of pivotal genetic determinants of CS that could be leveraged in future breeding programs. While a response specificity to CS has been demonstrated in a large range of studies (Mantri et al., 2010; Atkinson et al., 2011; Prasch and Sonnewald, 2013; Kissoudis et al., 2014; Ramu et al., 2016; Sinha et al., 2022), more comprehensive insights could be helpful in identifying generic components within those specific features as a game-changing step towards new strategies for the improvement of crops resistant/tolerant to fluctuating environmental challenges combining multiple constraints. The potential of such knowledge is unequivocal and particularly promising to provide effective genetic solutions for crop adaptation in multifaceted environments. Challenges that still remain in identifying the main components of CS specific responses in combined biotic and abiotic stresses as well as opportunities in identifying a generic compound of specific CS responses will be discussed in this opinion paper.

## When conditioning overshadows specific combined stress responses

### Not just a third-party issue

**Figure 1:**
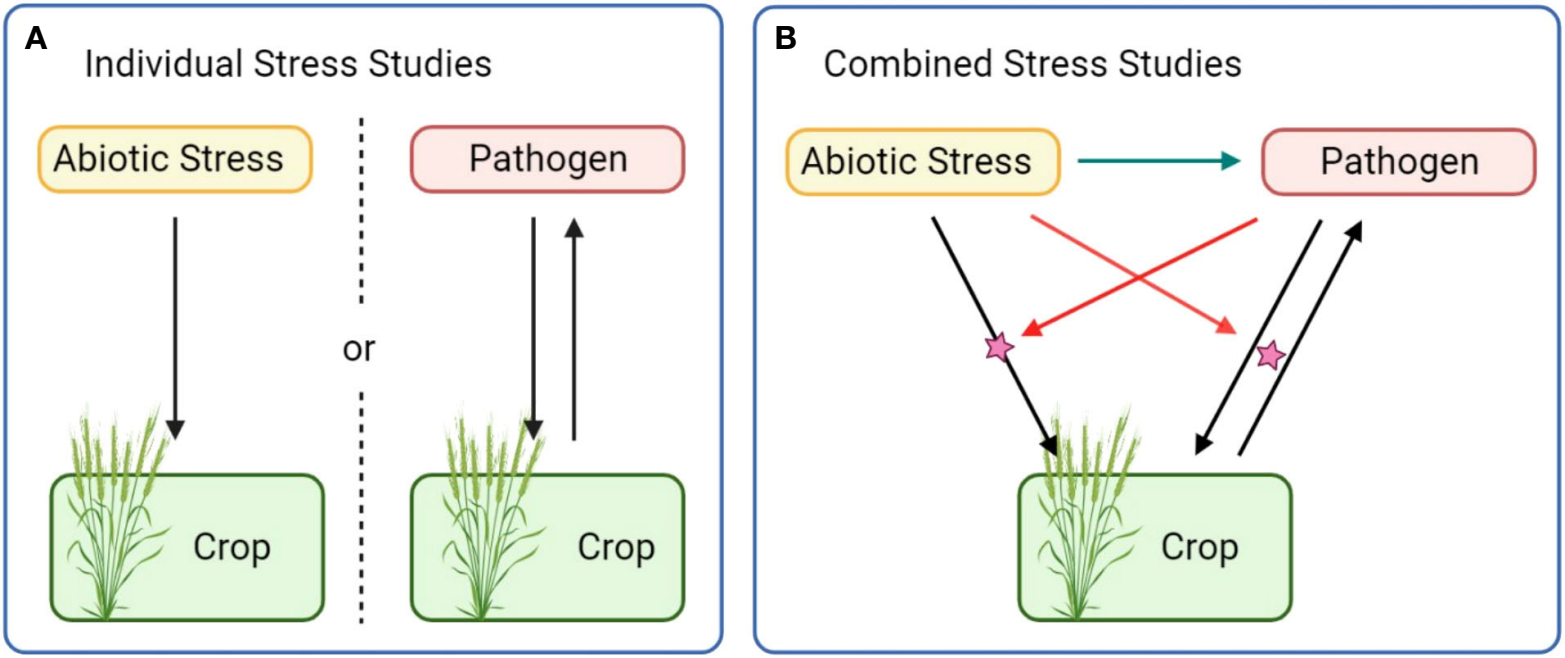
In single stress studies **(A)**, the effect of stressors is dissected independently of other sources of variation. Holistic assessment of combined stress **(B)** requires to account for a supplementary stressor and supplementary effects (colored arrows).

Theoretically, CS studies with the most straightforward conception aim to analyze a tripartite interaction between a plant, a first and a second stressor. From an experimental and statistical point of view, extraction of dual effects appears to be more complicated than dealing with individual stress data and as such, CS must be considered as a new composite stress. Disentangling the contribution of each effect of the CS and determining all contrasts from individual stress interactions remains complex and classical approaches systematically require to refer to single stress studies as comparisons. With an objective of combination evaluation, the most coherent way to fully characterize those tripartite interactions would be the evaluation of each bipartite interaction under the influence of the third actor (**Figure 1**). In other words, this suggests to not exclusively analyze the plant response to stress A in presence of B, but to also characterize the response to stress B in presence of stress A before concatenating all information and conclude about the combination and possible interactions. This requires to dissect the influence of a new player on the interaction balance, shifting our view of single plant-stressor interactions where the sole effect of one actor on another is usually emphasized.

Most of the CS studies described so far have dealt with only one bipartite interaction in the light of a third and varying entity, whereas the other interactions have been left aside. In combined biotic and abiotic examinations of crops, studies predominantly focused on the interaction between the plant and the biotic stressor under different abiotic environments while possible direct effects of the abiotic environment on the biotic stressors were mostly missed out. Even though assessing the complete CS response implies more modalities and is thus more fastidious, including all actors and interactions would probably help integrative understanding of CS.

### Multiplying constraints on crops and researchers

Managing two instead of one stress might require even more efforts as intended, because it opens inconspicuous questions. For instance, when should the constraints be applied? Although clearly challenging the prioritization between “observed” and “expected”, the answer seems to be absent from current literature where experiments vary from virtually simultaneous application starts of the stress conditions (e.g. Fu et al., 2009; Ramu et al., 2016; Farahbakhsh et al., 2019) to a delay of several weeks (Pennypacker et al., 1991; Rodriguez-Algaba et al., 2019). If stresses are applied sequentially rather than strictly simultaneous, the subsequent inquiry is the order of applied stressors. This is a particularly important point because modifying the order of stress onset can substantially change the plants response to the stress combination (Ramegowda and Senthil-Kumar, 2014). Consulting existing CS studies on crops since the 90s, the order systematically depends on the focal bipartite interaction emphasizing that most of the analyses have questioned CS mainly through the dissection of a given stress response in the prism of a second constraint. When plant tolerance to abiotic constraints is the focus, plants are most often first subjected to biotic stress (e.g. Xu et al., 2008; Davis et al., 2015). Contrarily, when studying resistance to biotic stress, the contrasting abiotic conditions are commonly established first (Dossa et al., 2020; Naz et al., 2021). Some earlier studies on plant responses to biotic constraints under different environments applied the biotic stress first (Martens et al., 1967; Pennypacker et al., 1991; Judelson and Michelmore, 1992). Depending on their nature, stresses can be established more or less rapidly and responses can be immediate to strongly delayed. In some cases, a disproportionate delay between stress application and the implementation of responses might be observed, leading to the exclusion of one of the constraints. This emphasizes that such delays should be considered for an appropriate experimental design able to reflect the expected environmental changes including likely climate irregularities and their consequences on biotic interactions. Another critical question is the magnitude of each stress intensity which is first assumed not to directly prevent the installation of the other stress and secondly supposed to be comparable between constraints of different nature. This has been thoroughly reviewed by Desaint et al. (2021) providing nice examples of how temperature increase can condition different responses to pathogens.

### Sorting out CS responses among many-sided variables

One of the major difficulties in evaluating CS responses is the choice of relevant variables which can specifically characterize the concurrent action of the stressors. Assessed at different scales, ranging from the anatomical (Wiese et al., 2004), cellular (Khasin et al., 2021), physiological (Chekali et al., 2011), metabolic (Cohen et al., 2017; Christensen et al., 2021), genetic (Choi et al., 2013; Vemanna et al., 2019) to the epigenetic level (Rodriguez-Algaba et al., 2019), they usually consist of variables that perfectly characterize single stress conditions but neglect specific cross-effects of stress combinations. Variables shared between stresses constituting the combination, such as phytohormone balance or oxidative markers, are suggested (Pandey et al., 2017; Anwar et al., 2021) but *bona fide* parameters still have to be compared between individual, combined or sequential stresses. The specificity of CS responses in crops is not restricted to a particular situation or to the amplitude of the responses but there are also unique characteristics and traits reported in CS that are absent in the corresponding single stresses. This observation strongly suggests that CS needs to be considered as new and unique stress. Accordingly, integrative variables might be needed evaluating stress combinations overcoming the difficulties in choosing variables mentioned above. Focusing on a variable that is commonly modified in limiting conditions would be a good indicator for comparisons between stresses. A feature that is affected by all isolated stresses is *per definitionem* the plant’s “fitness”. While the response of stress combinations on this variable can differ in direction and amplitude, its evaluation would be interesting because the outcome of the combination becomes rapidly evident. Since decades, yield is used as such an indicator of reproduction closely connected with economic concerns but we are convinced that evaluating the viability and growth of crops could be preferred for research purposes. Universal variables like photosynthesis, leaf growth and reproduction are easily accessible, non-destructive and can give straightforward information about the interaction effects between constraints.

## Leveraging genericness in unique CS responses towards new multi-purpose determinants for the challenges of global change

Despite increasing evidence for unique responses, a generic part could be shared in the specific responses of CS. A way to identify possible genericness could be the use of holistic approaches such as the different -omics studies already suggested for other reasons to be the future in the identification of unique CS responses (Pandey et al., 2015; Anwar et al., 2021). Large-scale -omics studies would not only enable to validate the hypothesis of generic components in specific CS responses but also allow the identification of the physiological processes and their primary regulators involved in that part of the response. The identification of CS-driven biological processes would allow to closely understand the plant strategy facing multiple constraints, while knowledge on their central regulators would be extremely valuable in crop breeding programs to leverage molecular markers of wide adaptive traits with possible broad-spectrum effect on different and co-occurring stresses. Versatile markers of shared specific responses would probably allow to further decomplexify CS studies by lessening the observation variables. A major criticism on current and past CS studies on crops is to neglect the permanently changing conditions encountered in the field which become even more fluctuating in the context of climate change. Efforts should also be refocused on the experimental modalities being tested e.g., consideration of increasing environmental variability and realistic scenarios responding to current climate change problematics could be investigated. So why not profit from realistic field conditions in order to broaden our knowledge on relevant CS issues? Chilakala et al. (2022) for instance used complementary pot and field experiments bringing light in how drought and heat stress affect dry root rot disease development. Of course, less controlled conditions can notably increase the complexity of the studied system and therefore the analysis of such experiments but powerful approaches as the different -omics and systems biology tools are available. We probably need to rethink how to use them and eventually adapt predictive biology and modeling strategies to our CS problematics in order to take advantage of such naturally existing environmental complexity. Original attempts from phenotypic reports and weather factors have already been successful such as in Sinha et al. (2021), who used correlation analysis and artificial neuronal networks to disentangle complexity in field and succeeded to predict dry root rot disease in chickpea from simulation modeling. Further, finding generic compounds in uniquely expressed CS responses requires to design and organize databases gathering different CS studies that could be compared. A CS platform such as SCIPDb (Priya et al., 2022) could open new avenues in identifying conserved genetic and molecular determinants of CS. However, one should be cautious about the comparability of studies accounting for often heterogeneous methodology and plant models. Compiling different but complementary approaches might become important and certainly systems biology will play a leading role, especially in describing how shared and stable the regulation networks of CS responses and their master regulators are.

Overall, CS has been studied for several years, although its complexity is not yet disentangled. A lever to disentangle CS complexity could be an approach taking advantage of the natural occurring complexity as expected in field and the adaptation of available tools to those conditions. It remains keen but will be important in the future to see the environment in its entirety as well as enlarge the biotic constraints to a consideration of the plant holobiont influence on the answers to a complete and non-static abiotic environment. Promising achievements of plant performance and bacterial composition have already been described under natural environments (Flemer et al., 2022), showing the power of available tools smartly employed.

## Author contributions

All authors contributed to the article and approved the submitted version.
